# Family joint activities in a cross-national perspective

**DOI:** 10.1186/1471-2458-7-94

**Published:** 2007-05-30

**Authors:** Apolinaras Zaborskis, Nida Zemaitiene, Ina Borup, Emmanuel Kuntsche, Carmen Moreno

**Affiliations:** 1 Institute for Biomedical Research of Kaunas University of Medicine, 4, Eiveniu str., Kaunas, LT-50009, Lithuania; 2 Nordic School of Public Health, Box 12133, SE-402 42 Göteborg, Sweden; 3 Swiss Institute for the Prevention of Alcohol and Drug Problems, Research Department, P.O. Box 870, CH 1001 Lausanne, Switzerland; 4 University of Sevilla, Dept of Developmental and Educational Psychology, Faculty of Psychology, 41018 Sevilla, Spain

## Abstract

**Background:**

Parents and children joint activities are considered to be an important factor on healthy lifestyle development throughout adolescence. This study is a part of the Cross-National Survey on Health Behaviour in School-aged Children – World Health Organization Collaborative Study (HBSC). It aims to describe family time in joint activities and to clarify the role of social and structural family profile in a cross-national perspective.

**Methods:**

The research was carried out according to the methodology of the HBSC study using the anonymous standardized questionnaire. In total, 17,761 students (8,649 boys and 9,112 girls) aged 13 and 15 years from 6 European countries (Czech Republic, Finland, Greenland, Lithuania, Spain, and Ukraine) were surveyed in the 2001–2002 school-year. The evaluation of joint family activity is based on 8 items: *(1) *watching TV or a video, *(2) *playing indoor games, *(3) *eating meals, *(4) *going for a walk, *(5) *going places, *(6) *visiting friends or relatives, *(7) *playing sports, *(8) *sitting and talking about things (chatting).

**Results:**

Students from Spain and Ukraine reported spending the most time together with their families in almost all kinds of joint activities, whereas students from Greenland and Finland reported spending the least of this time. Boys were more likely than girls to be spending time together with family. Joint family activity goes into decline in age from 13 to 15 years. Variability of family time in a cross-national perspective was relatively small and related to children age category. Considering national, gender and age differences of studied population groups, we found that the distribution of joint family activities tends to be dispersed significantly by family structure (intact/restructured family) and family wealth.

**Conclusion:**

Our study compares children and parent joint activities in European countries and reveals differences and similarities in these patterns between countries. The findings underline the role of family structure (intact/restructured family) and family wealth in the distribution of time spent in joint family activities, which should be considered by health promoters.

## Background

Family time, or parents and children spending time together in joint activities, is a dimension of successful families related to communication and often measured in terms of families rituals, including family mealtime [1,2]. Joint family activities contribute to the well-being of each family member and enhance quality of communication between family members [3].

Different studies prove positive effects of family time on overall children development, school achievements and future career [4-6]. Family time has shown strong and consistent relationship with smoking, drinking, drug intake, sexual intercourse and pregnancy, as well as delinquent behaviour of children [7-10]. Parents are attached to their children, relate to them, resolve conflicts in particular ways, and throughout common activities transmit patterns of behaviour. Children in their turn are attached to their parents, love them, fight with them, do things together and the quality of this primary social bond is very influential on children's well being and problem behaviour [11,12]. In addition, it has been established in several studies that attachment to parents has the strongest influence on emotional well-being and emotional problems [11]. Generally, in childhood family life may have more direct effects on health than material factors and, through social mobility, may be indirectly linked to health inequalities in adulthood [7].

Adolescence is typically described as a time of diminishing parental influence. Adolescents tend to spend more and more of their leisure time with peers and less and less with their parents [13]. However, there are few data available on parents and children joint activities throughout adolescence, compared with early childhood, but these have shown that finding more time to spend with their children is a high priority for parents. For example, according to the recent survey of Alabama's parents, 53% of them believed they did not have enough time to spend with their children; however, two out of three parents stated that, if they had more time to spend with their children, they would not use this time watching television [14].

Nowadays families, as society itself, are affected by rapid changes. The idea that family time is disappearing is gaining growing attention because data show that parents are spending less time together as a family and especially on family meals, and even more so on working days [15].

An increase of single parent families has been observed in most European countries [16-18]. Recently, several studies have provided evidence that living in a single parent family and a low attachment to parents is associated with adolescent substance use and other health threatening outcomes [19]. However, it is still unclear whether family structure is related to joint family activities, and how consistent such relations are across European countries. The same applies to family wealth and other family matters that might be related with joint family activities. Therefore, it is important to investigate parents and children joint activities as well as their determinants in a cross-national perspective.

Recent time-use studies have shown that these concerns may have been a relevant issue for public health specialists as well [20]. Joint family activities are the most important indicator of family functioning. Therefore, focus on it is essential for the development of effective health education programs and practice targeted at young people.

A WHO research program, Health Behaviour in School-aged Children (HBSC), aims to gain new insight into, and increase understanding of, health behaviour, lifestyles and their context in young people [21-23]. A specific objective of this study was to collect data on family culture that include socio-economic characteristics of the family, parents and children's communication, parental monitoring, parental style of upbringing, encouragement of individuals and social support, and family time [24]. The data collected provide the participating researchers with opportunities to do cross-national comparisons of structural and process aspects of family life.

In regard to a cross-national perspective, the present study aims i) to describe parents and children joint activities, and (ii) to clarify the influence of family structure and family wealth on family time spent in joint activities. The samples obtained for this study represent 13 and 15-year-old schoolchildren from six countries of Europe (Czech Republic, Finland, Greenland, Lithuania, Spain and Ukraine) in the 2001–2002 school year.

## Methods

### Sample

The 2001/2002 Health Behaviour in School-Aged Children Study is a World Health Organization (WHO) supported study of nationally representative samples of adolescents in 36 countries and regions [21-24]. In each country, a cluster sample design was applied with school classes as sampling units. The specific populations selected for sampling include those in-school youth aged 11, 13 and 15. Schools and classes within schools were selected to be representative by age level and regional geography. Recommended sample sizes for each country were 1,536 students per age group. Sample sizes assured a 95% confidence interval of ± 3% for prevalence estimates, with a design factor of about 1.2 across countries [24].

The present analysis is based on 17,761 students (8,649 boys and 9,112 girls) aged 13 and 15 years from 6 countries: Czech Republic, Finland, Greenland, Lithuania, Spain, and Ukraine (Table 1). Students from only these countries answered the questions about family time spent together in shared activities that were optional to the standard HBSC questionnaire. For all participating countries response rates were over 90%. The 11-year-age group was excluded from the analysis, as students of that age in Finland did not complete the question on family time.

**Table 1:** Number of students who answered questions about family time, by country, gender and age category

Gender	Country	Age category		Total*
			
		13	15	
Boys	Czech Republic	780	806	1586
	Finland	873	867	1740	
	Greenland	155	100	255	
	Lithuania	954	981	1935
	Spain	991	821	1812
	Ukraine	591	730	1321
	**Total**	**4,344**	**4,305**	**8,649**

Girls	Czech Republic	881	854	1735
	Finland	845	874	1719
	Greenland	192	138	330
	Lithuania	919	923	1842
	Spain	974	935	1909
	Ukraine	706	871	1577
	**Total**	**4,517**	**4,595**	**9,112**

*Total number 17,761

### Measures and variables

Full descriptions of the questionnaire items employed and their development have been published in the protocol of the study [24]. National questionnaires are translations and adaptations of the international standard version, with independent re-translation back to English, to maximize comparability.

*Family time together *(spending time together in shared activities). The list of things which some families do together includes 8 items: (1) watching TV or a video together, (2) playing indoor games together, (3) eating a meal together, (4) going for a walk together, (5) going places together, (6) visiting friends or relatives together, (7) playing sports together, (8) sitting and talking about things together. Students were asked how often did they do any of these activities and spend time together in shared activities. The response categories were 'every day' (coded as 4), 'most days' (coded as 3), 'about once a week' (coded as 2), 'less often' (coded as 1), and 'never' (coded as 0). For each variable, missing values were replaced with the variable mean. The following five indicators of the family structure and wealth were considered as factors that might influence family time.

#### (1) Parents

The survey used a set of items that interest most countries and that reflect general cross-national family systems. The question on family structure allowed children living in more complex family set-ups to answer for two homes: the first for main or only home and the second other home if a child under family circumstances lives a substantial time outside his or her main home ([23], p.27). The respondents were asked to identify people with whom they live in each home: parents, stepparents, grandparents, children or other adults.

For the purposes of this article, family structure was assessed only for the main home. A variable PARENTS designed for our analyses was coded into four categories: both natural parents (intact family), single parent (one natural parent and no step-parent), stepfamily (restructured family; one natural parent and a step-parent were present), other (neither natural parent present). In our study, analyses were limited to children living in households of the first three categories; the group of the fourth category respondents was excluded from the analyses because there were too few children living in other family structures for meaningful inference (2.6% of children overall).

#### (2) Grandparents

Based on the list of persons who lives in the main home, a variable GRANDPARENTS states if any grandparent lived there or not.

#### (3) Children

For the main home, respondents were asked to write down how many brothers and sisters lived there (including half, step or foster brothers and sisters). A variable CHILDREN was constructed with three categories: neither brothers nor sisters, one or two brothers or sisters, three or more brothers and sisters.

#### (4) Home

In our pilot studies, we found that quite a number of children might have two homes (not including holiday or summer houses), especially when parents are not living together. As well as listing the people in each home, students were also asked to indicate the amount of time they spend there. A variable HOME that was used in the present analyses indicates time in main home and have three categories: all the time, most of the time, half the time.

#### (5) Family wealth

The family affluence scale (FAS) is developed for HBSC surveys as a measure of family wealth ([23], p.15). It comprises four items, which young people are likely to know: family car ownership, bedroom occupancy, family holidays, and computer ownership. A composite FAS score was calculated for each young person based on his or her responses to these four items. For the analyses, we used a three-point ordinal scale, where FAS = 1 indicated low affluence; FAS = 2 indicated middle affluence, and FAS = 3 indicated high affluence.

### Statistical analysis

Basic and advanced statistics procedures of the Statistical Package for Social Sciences (SPSS) for Windows version 13.0 software package were used to conduct data analysis. The first analyses included descriptive statistics, primarily frequencies, provided understanding distributions of the respondents' gender, age, country, family structure, family wealth and variables of family time spending together in shared activities. Chi-square test, with the significance level 0.05, was applied to access difference in distributions of these variables between groups of respondents.

In order to assess family time in general, all the above-mentioned eight family activity items were combined into one derivative variable labeled "Family Time Index" (FTI) [10]. It was not the simple sum of scores in responses to each of the 8 questions, but rather a linear combination of them with different weights. The values of weights in such linear combination were estimated from the total data set of all participating countries that includes 17,761 records by the SPSS Factor Analysis procedure requesting a single factor, and then calculating and saving that single factor (FTI) for each record [25]. The values for FTI appeared to be distributed within the range of -2.566 and 3.545; its mean and standard deviation estimates, as follows by definition, were 0 and 1 respectively. Positive FTI values were typical of the families where the members reported more commonly-spent time; and, vice versa, negative FTI values demonstrated that families tended to spend less time together. Weights of variables in its linear combination (factor score values), as indicators of variables involvement in a factor, reflect the partial variable variances in the factor variance. The percentage of the total variance of all 8 items explained by the FTI was 41.16.

The last analyses included analysis-of-variance for the FTI variable by 3 covariates (gender, age and country) and 5 factors of family structure and wealth. These analyses were performed using GLM Univariate Analysis Procedure [25]. To examine our hypothesis that family time might be affected by family structure and family wealth we tested three consecutive models. First (Model A), a model that includes only three covariates: gender, age and country. Second (Model B), a model that includes 5 fixed factors (the items of the family structure and family wealth as predictors or independent variables). Third (Model C), a model with all the significant predictors from the preceding analysis adjusted by gender, age and country. The last model also included interaction terms between country and all fixed factors in order to analyze the country specific role of family structure and family wealth on shared activities (interactions between covariates are excluded by sampling procedures). In multiple comparisons of observed means homogeneous subsets of data were defined by Bonferroni test for Post Hoc analysis.

## Results

### Descriptive Results

**Figure 1:**
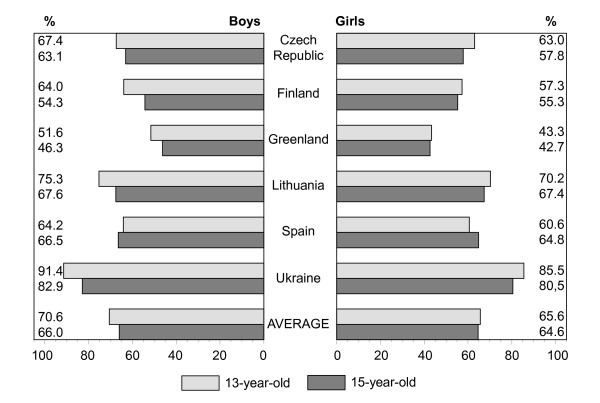
Percentage of students who watch TV or video together with family every day or most days, by country, gender and age.

**Figure 2:**
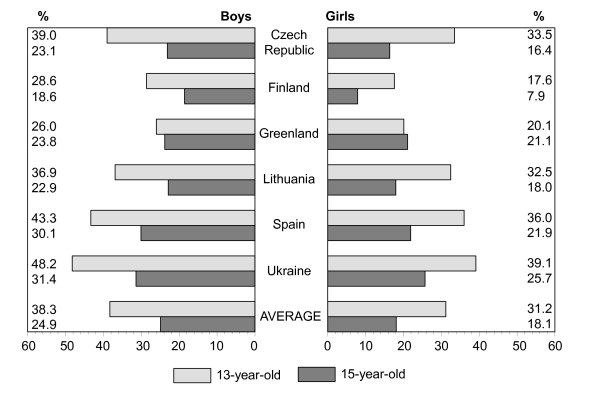
Percentage of students who play indoor games together with family every day, most days or about once a week, by country, gender and age.

**Figure 3:**
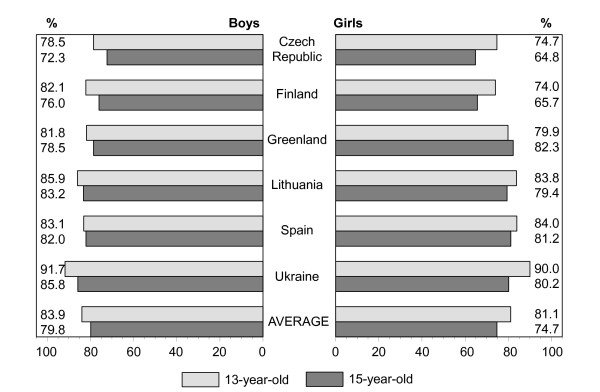
Percentage of students who eat a meal together with family every day or most days, by country, gender and age.

**Figure 4:**
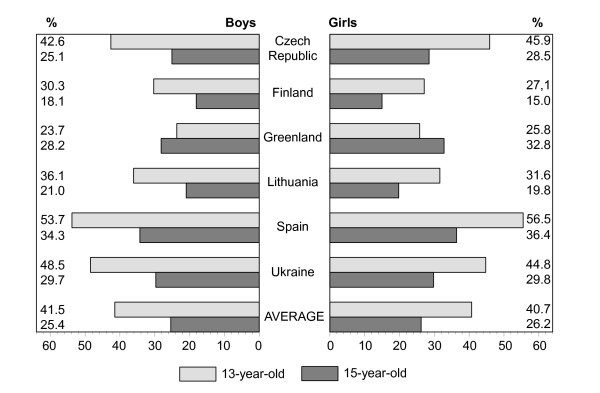
Percentage of students who go for a walk together with family every day, most days or about once a week, by country, gender and age.

**Figure 5:**
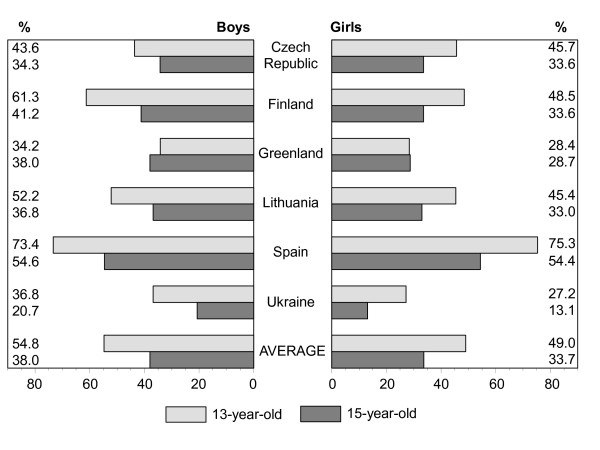
Percentage of students who go places together with family every day, most days or about once a week, by country, gender and age.

**Figure 6:**
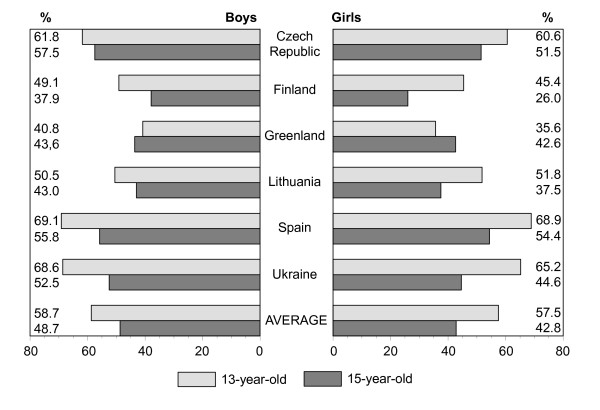
Percentage of students who visit friends or relatives together with family every day, most days or about once a week, by country, gender and age.

**Figure 7:**
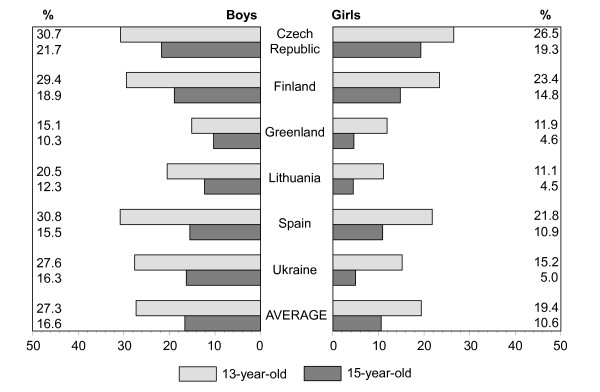
Percentage of students who play sports together with family every day, most days or about once a week, by country, gender and age.

**Figure 8:**
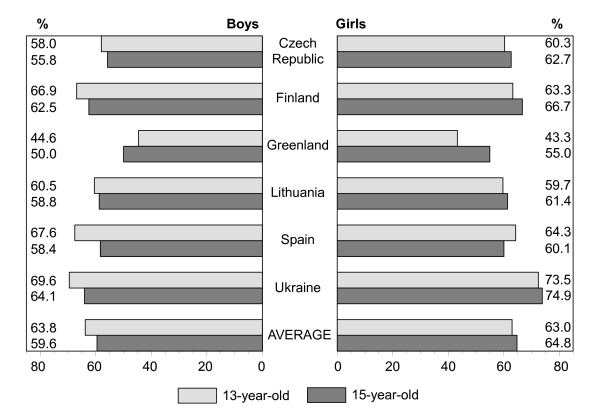
Percentage of students who sit and talk about things together with family every day, most days or about once a week, by country, gender and age.

Figures 1, 2, 3, 4, 5, 6, 7, 8 show how many children spend time together with family in different shared activities, by countries, gender and age group. From the figures shown it is difficult to generalize that students from several countries tend to spend more or less family time, but looking into the histograms allows to find out that students from Spain and Ukraine reported *most often *and students from Greenland and Finland reported *most rare *about spending regular time together with family in many kinds of joint activities.

In regard to gender and age differences, the same consistent ranking patterns can be found across most of the countries. For both boys and girls, the proportion of students who reported spending time together with family in joint activities on a regular ('every day', 'most days' or 'about once a week') basis declined as the age of the respondents increased. In both age groups, boys were more likely than girls to spend time together with family in joint activities, especially in playing sports, playing indoor games, watching TV or video, going places. Students' responses on sitting and talking about things with their families is an exception to that rule because 15-year-old girls were more likely than boys and more likely than 13 year old girls (with the exception of girls from Spain) to spend time together with their families sharing this kind of activity.

### Family Time Index

The results presented in Table 2 show that eating a meal together, watching TV or a video together, sitting and talking about things together are among the most frequent joint activities, while playing sports together is the less frequent activity. The standard deviations fell in the range 0.916 to 1.260; this quite narrow range indicates that there were no conspicuous deviations in variance of family time items.

**Table 2:** Means and standard deviations of responses^a ^to questions on family time spent together in shared activities and corresponding factor score values

Shared activity	Mean^b^	Standard Deviation	Factor Score Values^c^
Watching TV or a video together	2.78	1.116	0.147
Playing indoor games together	1.12	1.011	0.203
Eating a meal together	3.15	1.053	0.154
Going for a walk together	1.23	1.007	0.237
Going places together	1.53	0.978	0.229
Visiting friends or relatives together	1.67	0.916	0.205
Playing sports together	0.78	0.978	0.191
Sitting and talking about things together	2.12	1.260	0.193

^a ^Response codes are: every day = 4, most days = 3, about once a week = 2, less often = 1, never = 0.

^b ^For each variable, missing values are replaced with the variable mean.

^c ^Extraction method: principal component analysis. Percentage variance explained by factor 41.16; Kaiser-Meyer-Olkin measure of sampling adequacy 0,845; Bartlett's test of sphericity: approx chi-square 30,509; df 28; sig. 0.000.

Correlations between family time items were checked next. Correlation coefficient values varied from 0.119 to 0.564; they were all significant at the 0.01 error level. The lowest correlation value was between 'watching TV or video together' and 'playing sports together'; the highest correlation value was shared by 'going for a walk together' and 'going places together'. Additionally, internal consistency of the family time scale items (Cronbach's alpha) was equal to 0.786.

Factor score values, which are presented in Table 2, show weights of responses to each of the 8 questions on family time in construction of the FTI value. There is a light difference in weights of responses on family time. The greatest weights have items 'going for a walk together' (0.237) and 'going places together' (0.229). High factor score values show that these family time items are extensively correlated with other items and consequently have a big impact on the FTI variance. The smallest weight (0.147) was that of 'watching TV or a video together' which has a weak interrelation with other family time items.

### Determinants of family time spent together

The last objective was to examine whether the factors of family structure and family wealth make distinct contributions to explaining the variation of family time spent in joint activities. However, before the possible interrelations between these factors are considered, it is necessary to explore to what extent does the variation of family time in joint activities depends on country, gender and age.

Table 3 shows FTI mean values variation across countries in gender and age groups. To explore these data it is important to have in mind that positive FTI values are typical of the families where children reported spending more family time together, and negative FTI values demonstrated that families tended to spend less time together. There were statistically significant differences in FTI mean values between countries. Conspicuous variance of FTI means between countries was observed in the group of 13-year-olds. Both for boys and girls, the most positive values were observed among students from Spain and Ukraine, while the most negative values were in students from Greenland. In the group of 15-year-old boys Spain and Ukraine were also the countries in which the boys could be characterized as more often spending time in common family activities, but in the opposite end was Finland, Czech Republic and Lithuania. In girls of the same age only the Spanish remained as having the greatest tendency to spend time together while the Finns and the Lithuanians had the most negative assessments. Such ranking of FTI mean values summarizes the findings that might be obtained from the analysis of individual family activities (see figures).

**Table 3:** Means (stand. error) of the Family Time Index by country, age and gender. Univariate and Post Hoc analysis^a^

	13-year-olds		15-year-olds	
	
	Boys	Girls	Boys	Girls
Czech Republic	0.136 (0.034)	0.105 (0.031)	-0.195 (0.030)^#^	-0.236 (0.027)
Finland	0.169 (0.031)	-0.021 (0.031)	-0.207 (0.029)^#^	-0.357 (0.029)^#^
Greenland	-0.179 (0.071)^#^	-0.289 (0.068)^#^	-0.151 (0.086)	-0.192 (0.071)
Lithuania	0.188 (0.038)	0.009 (0.037)	-0.186 (0.033)^#^	-0.333 (0.031)^#^
Spain	0.454 (0.035)*	0.392 (0.035)*	-0.014 (0.034)*	-0.099 (0.032)*
Ukraine	0.438 (0.040)*	0.250 (0.037)*	-0.058 (0.035)*	-0.198 (0.028)
AVERAGE	0.256 (0.016)	0.130 (0.015)	-0.137 (0.014)	-0.242 (0.013)

^a ^Bonferroni test: * maximal mean value or no statistical difference from the maximal mean value; ^# ^minimal mean value or no statistical difference from the minimal mean value.

A small, but nevertheless, statistically significant difference in mean values was detected between boys and girls indicating that boys (0.061) are more likely than girls (-0.058) to spend time together with their families. Comparison of the mean values of FTI between age categories shows considerably more family time spent together among 13-year-olds (0.192) than among 15-year-olds (-0.191).

Mean values of the FTI were compared across categories of family structure and family wealth (Table 4). In comparison with children in two-parent families (intact families), children of single parents and, especially, children of restructured families indicated less frequent joint activities in their families as seen in significantly negative means of FTI values. Grandparents' presence or absence in the family does not play a significant role in spending family time together. Adolescents from families with many children (3 or more siblings) reported spending less time in joint family activities than adolescents from families having only one child.

**Table 4:** Descriptive statistics and univariate analysis of Family Time Index variance, by family structure and family wealth variables

Variables*		N	Mean	Standard Deviation	Standard Error	95% Confidence Interval for Mean		Significance (F test)
							
						Lower Bound	Upper Bound	
PARENTS:	Both parents	13,459	0.043	0.985	0.008	0.026	0.060	<0.001
	Single parent	2,291	-0.059	1.047	0.022	-0.102	-0.016	
	Stepfamily	1,551	-0.190	0.979	0.025	-0.239	-0.141	
GRAND-PARENTS:	Absent	15,008	-0.001	0.989	0.008	-0.017	0.015	0.703
	Present	2,753	0.007	1.060	0.020	-0.033	0.046	
CHILDREN:	No brothers and sisters	2,455	0.025	0.991	0.020	-0.015	0.064	0.002
	1–2 brothers or sisters	12,558	0.014	0.983	0.009	-0.004	0.031	
	3 or more brothers or sisters	1,876	-0.069	1.044	0.024	-0.116	-0.023	
HOME:	All the time	13,223	0.039	0.999	0.009	0.022	0.0556	<0.001
	Most of the time	2,997	-0.057	0.963	0.018	-0.091	-0.022	
	Half the time	1,087	-0.286	1.028	0.031	-0.347	-0.225	
FAS:	Low	7,128	-0.167	0.996	0.012	-0.190	-0.143	<0.001
	Middle	7,268	0.087	0.983	0.012	0.064	0.110	
	High	2,993	0.186	0.986	0.018	0.151	0.221	

* PARENTS – parents and step-parents in household; GRAND-PARENTS – grand-parents in household; CHILDREN – number of brothers and sisters; HOME – time living in main home; FAS – Family Affluence Score.

Considering the fewer amount of time students spend in main homes, there was an evident negative impact of that factor on family joint activities. The family wealth gradient had a significant relationship with the FTI indicating that children in highly affluent families are able to spend more time together with family than their peers in less affluent families.

Table 5 displays results from analysis-of-variance and shows tests of between-subjects effects on index of spending family time together in shared activities. The following findings were set out.

**Table 5:** Tests of between-subjects effects. Dependent variable: Family Time Index. Given are main-effects and interactions between variables* in the models

Source	Sum of Squares	df	Mean Square	F	Sig.	Partial Eta Squared
**Model A**	442.0	3	147.4	151.1	<0.001	0.025
COUNTRY	381.0	1	381.0	390.7	<0.001	0.022
GENDER	5.9	1	5.9	6.1	0.014	<0.001
AGECAT	223.8	1	223.8	229.5	<0.001	0.013
Error	17318.0	17758	1.0			
Total	17760.0	17761				
**Model B**	470.5	9	52.3	55.3	<0.001	0.030
FAS	289.4	2	144.7	153.0	<0.001	0.019
HOME	90.5	2	45.3	47.9	<0.001	0.006
CHILDREN	6.4	2	3.2	3.4	0.035	<0.001
PARENTS	47.0	2	23.5	24.8	<0.001	0.003
Error	15157.1	16022	0.9			
Total	15627.6	16032				
**Model C**	1221.8	22	55.6	67.9	<0.001	0.078
FAS	46.4	2	23.2	25.8	<0.001	0.003
HOME	16.5	2	8.3	9.2	<0.001	0.001
CHILDREN	9.9	2	5.0	5.5	0.004	0.001
PARENTS	6.5	2	3.2	3.6	0.027	<0.001
COUNTRY	21.0	1	21.0	23.4	<0.001	0.001
GENDER	36.3	1	36.3	40.3	<0.001	0.002
AGECAT	534.4	1	534.5	593.9	<0.001	0.036
FAS * COUNTRY	3.3	2	1.7	1.9	0.157	<0.001
HOME * COUNTRY	17.7	2	8.8	9.8	<0.001	0.001
CHILDREN * COUNTRY	7.0	2	3.5	3.9	0.020	<0.001
PARENTS * COUNTRY	3.5	2	1.7	1.9	0.147	<0.001
Error	14405.8	16012	0.9			
Total	15627.6	16032				

* GENDER – gender; AGECAT – age category; COUNTRY – country; HOME – time living in main home; PARENTS – parents and step-parents in household; CHILDREN – number of brothers and sisters; FAS – Family Affluence Score.

Model A shows that all three covariates (country, gender and age), when entered together, are significant predictors explaining the variation of the FTI value, even if the contribution of gender was quite low in comparison with country and age variables.

Model B sets out significant findings when analysis-of-variance was run with 4 fixed factors that include family structure and family wealth items only. Number of children in the family has the smallest impact on variation of the FTI value while the FAS has the highest significance.

Model C includes the same fixed factors as model B, however, adjusted by gender, age and country. Possible interactions between country and all fixed factors were considered too. This model reveals that most of the FTI variation is due to age category; significant main-effects of FAS and HOME variables are as high as those of COUNTRY and GENDER variables; only two interaction terms, HOME*COUNTRY and CHILDREN*COUNTRY, have a significant effect at least at the 5% error level.

## Discussion

The present study examined the amount of time parents and children spend in joint activities based on national representative samples from six European countries (Czech Republic, Finland, Greenland, Lithuania, Spain and Ukraine) and its relationship with family structure and family wealth. Participating countries are located in different geographical regions of the continent and are characterized by different socio-economic status and historical and cultural experience on family-demographic affairs. From a cross-national point of view, our study is the first study of this kind. The present study does not aim to precisely reflect the actual family process, but points out usual daily routines and free time activities shared by family members.

The results of the present study have revealed both strong similarities and striking differences between countries concerning parents and children family joint activities.

In all countries, 13-year-old children were more likely to be involved in activities shared with their parents in comparison to 15-year-olds. In Western cultures, adolescence is typically a time of diminishing parental influence and growing peer influence [13,26,27].

Similarly, boys were more prone to spend time together with their families, than girls. Such gender difference can be influenced by the type of the activities chosen to evaluate family time. Most of them tend to be more attractive to boys. Different studies provide the evidence that boys are significantly more involved in physical activities than girls, while girls spend more the time in social activities compared with boys [28,29]. These statements support our findings.

Cross national data comparison shows that Spain and Ukraine stand out as an extreme case with its high positive FTI, which means that students in these countries a higher amount of time were spending in joint family activities than in other countries. Greenland and Finland stand out at the opposite end of the scale by the same criterion. However, it is difficult to explain why Ukraine has higher scores that Lithuania and why both countries have higher scores than Czech Republic and Finland. These family processes could be expressions of cultural and socio-demographic influences. From Finland, for example, it is likely that due to the good childcare system most women have the possibility to go to work, whereas that appears not to be the case in Spain. Apart from this, (see below) Spain has a rather low divorcement rate while Finland's is rather high.

Family routine and leisure time in joint activities was also examined from its socio-economic perspective, assessing family structure and family wealth.

Radical social changes in household formation over the past decades have given rise to the single parent family in most European countries [16-18]. A number of recent studies indicate that single parents have restricted time to spend together with their children compared to two-parent families [30,31]. Because of strong associations between poverty and family structure [32], we suppose that single parents have to work more to support their children and thus have less time for joint activities with their children.

We compared the results of the present study with demographic data on family structures in different European Countries. The recent Fertility and Family Survey of 16 countries reported the fractions of time that children typically spend with both their original/biological parents, with a lone/single mother and in a stepfamily [33]. Findings from 4 countries of this survey (Czech Rep., Finland, Lithuania and Spain) might also be helpful for our study. The survey has highlighted the oneness of Spain, which stands out as a country where practically all children are born in married families and very few of them have experienced dissolution of their parents' union before they turn 15. These findings were proved by results of the HBSC survey of 2001/2002 [23]. The last results show that 86.7% of Spanish students live with both parents, while in Greenland this figure was 62.2% (these percentages correspond to the maximal and to the minimal values among countries which participated in our study). Although the associations between variables at the ecological studies do not necessarily represent the associations that exist at the individual level, data on family structures from above-presented studies, however, correlate with family time together and could be helpful to explain its variability across countries.

Our analysis have demonstrated that there is sufficient evidence to merge all family time items by the SPSS factor analysis process to form one factor that was named Family Time Index (FTI) [10]. This approach has shown how family time spent together in shared activities varies between different European countries as well as across age, gender and family structure and wealth groups. Multivariate analysis of variance has revealed that cross-national variability appears to have a minor effect compared to other variables in our study; most of the family time variability is due to age category. Considering national, gender and age differences of studied population groups, we found that the distribution of joint family activities tends to be dispersed significantly by family structure (intact/restructured family) and family wealth.

Our results increase understanding of family process during adolescence and might help health promoters working with school-aged children families to develop programs, which would consider these differences.

## Conclusion

We believe that our cross-country comparison has provided a lucid overview of the actual family joint activities situation in Europe. It also reveals differences and similarities in patterns across countries. These family processes, we argue, are expressions of cultural and socio-demographic influences. However, variability in family time in a cross-national perspective was relatively small in comparison with variability related to children age and gender categories in all populations. Considering national, gender and age differences of studied population groups we found that the distribution of joint family activities tends to be dispersed significantly by family structure (intact/restructured family) and family wealth too. However, further research is needed in order to assess the associations between family time together and health behaviour of adolescents. All these may assist the public health authorities in developing health promotion strategies, which will efficiently tackle these health issues early in life.

## Competing interests

The author(s) declare that they have no competing interests.

## Authors' contributions

AZ coordinated the survey in Lithuania, performed the statistical analysis, conceived and drafted the first outline of the paper. NZ outlined the study questions, substantially contributed to the conception and the design of the article and to the interpretation of data. IB, EK and CM provided important intellectual content. All authors read and approved the final manuscript.

## Pre-publication history

The pre-publication history for this paper can be accessed here:


